# River valley urban network and morphology: A study on the urban morphology evolution of Lanzhou

**DOI:** 10.1371/journal.pone.0302686

**Published:** 2024-05-28

**Authors:** Minan Yang, Yongsheng Qian, Xin Li, Yueqing Ou, Junwei Zeng

**Affiliations:** 1 School of Traffic and Transportation, Lanzhou Jiaotong University, Lanzhou, China; 2 School of Architecture and Urban Planning, Lanzhou Jiaotong University, Lanzhou, China; Al Mansour University College-Baghdad-Iraq, IRAQ

## Abstract

The present study investigates the dynamic evolution characteristics of urban spatial morphology by analyzing real road network data from 2000, 2010, and 2020, along with nighttime lighting data employing spatial analysis methods and spatial syntax models. Accordingly, two separate dimensions of urban morphology: internal and external, are covered. First, the integration and synergy of interior morphology features are analyzed using spatial syntactic modeling. Subsequently, the spatial compactness, fractal dimension, and level of center of gravity shift of the city are assessed by combining the nighttime lighting data with the earlier dataset. This analysis facilitated the deep exploration of the spatiotemporal evolution of the city’s external morphology. Building upon this foundation, the interaction between the "internal and external" domains was analyzed further. The main findings of the study reveal a synchronous pattern of urban expansion throughout the evolution of urban spatial morphology. Furthermore, the urban form was observed to undergo a progressive transformation, transitioning from a "single core" morphology to a "primary and secondary double core" morphology. Over time, this development progressed and evolved into a "belt-like multi-core" structure. Additionally, the coupling characteristics further validate the relationship between the structure of the road network and the urban morphology in river valley-type cities. In particular, accessibility of dense and horizontally distributed transportation network was found to significantly influence the spatial development of these cities. As observed, the findings provides valuable insights into understanding the characteristics of internal and external associations regarding urban spatial patterns.

## Introduction

“Urban morphology”, in the context of urbanization, refers to the spatial representation of urban politics, economy, society, and culture [1]. It plays a crucial role in the internal spatial reconfiguration and shaping of cities, directly influencing the sustainable development of urban areas [2]. In recent years, there has been a transition in urban development from an incremental approach to a more comprehensive approach. As a result, there has been an increased focus on studying the internal optimization of urban spatial morphology. This includes examining the characteristics of different types of urban spatial morphologies, their expansion patterns, evolution of their morphologies [2–5], and role of various mechanisms [6]. During the evolution of urban spatial form, the transportation network is recognized as the primary factor that influences the changes in urban form [7]. It has a significant impact on the internal spatial organization, travel behavior, and the accessibility of transportation links [8, 9]. However, urban space has experienced a significant transformation in recent years as a result of several factors, including scarcity of land resources, escalating urban housing costs, and growing demands for shopping and transportation. Consequently, urban development has increasingly shifted from expanding outward to improving internal spatial organization and optimization [10]. This shift has resulted in enhancing the understanding of urban spatial form.

The relationship between urban road infrastructure and urban form is highly interdependent and complex [11]. In this context, urban morphology has a notable impact on the spatial organization of urban road networks to a certain degree. For instance, various land use (such as commercial, residential, and industrial) have distinct effects on the demand for and scale of road transportation [12]. Previous research has shown that urban areas with a high population density typically require more feeder roads and multi-level road systems to effectively meet transportation needs [13]. In contrast, cities characterized by a low population density can depend on wider roads and fewer interconnections to meet their transportation needs. Furthermore, the design of cities can also impact the arrangement of roads in order to encourage walking, cycling, and the use of public transportation as a way to reduce dependence on automobiles, mitigate air pollution, and ease traffic congestion [14]. Notably, a rational transportation layout and scale level can not only ease traffic congestion and improve accessibility [15] but also significantly influence the overall spatial arrangement of an urban area, effectively addressing the connection between the inner and outer parts of the city [16]. The external expansion space of a city is typically restricted by geographical features like mountains and rivers, leading to a scarcity of usable land. As a result, the available space within the city undergoes continuous shrinkage. In this context, the road traffic network facilitates the connection between internal and external spatial relations. The current study is centered on the efficient organization and enhancement of the city’s traffic network, encompassing its accessibility, centralization, and resilience structures. The complex interrelationship between urban road networks and spatial morphologies comprises the key factor in promoting sustainable urban development in the future. Therefore, investigating the relationship between the growth of urban road networks and spatial morphology is highly significant.

Urban form is the study and analysis of the physical and spatial features that define urban entities. Urban development involves the transformation of the urban form, primarily encompassing centralized or decentralized forms. In 2006, the esteemed architect Eero Saarinen conducted a comprehensive analysis of the architectural structure of several cities, such as Stockholm, Copenhagen, Hamburg, Munich, and others. Consequently, the idea of "organic sparsity" was introduced [17], demonstrating the existence of a decentralized and natural arrangement. Accordingly, researchers have conducted further studies to analyze the spatial and temporal development of spatial expansion by investigating various factors such as the speed of urban expansion [18], the intensity of urban expansion [19], fractal dimension [20], compactness [21], and the shift in the center of gravity [22]. Several factors impact the urban form, including the economy, land accessibility, population changes, employment patterns, transportation systems, and policy frameworks. These factors are often interconnected and sometimes contradict one another [23]. Moreover, these factors are susceptible to continuous change, ultimately contributing to the complex nature of urban form [24]. Additionally, numerous researchers specializing in urban transportation systems have found that public transportation plays a crucial role in driving urban development within road networks [25]. This is attributed to the strategic positioning of bus stations and linking them to the central business district and other important areas in the metropolitan region, thereby maximizing the efficient use of land resources. Furthermore, the strategic placement of residential centers, retail centers, offices, public facility spaces, and specific hospitals and schools within a walkable radius promotes the optimal development of urban socio-economic activities within the city [26]. Moreover, the implementation of this system enables the convenient transportation of urban residents, including a wide range of urban mobility options [27]. This observation can be understood as an indirect demonstration of how urban transportation systems affect the arrangement and changes in urban activities. Contemporary urban areas also face the challenges of swift urbanization and increased reliance on motor vehicles. In this context, most Chinese cities and towns are likely to adopt a trajectory of haphazard spatial expansion. Notably, the urban layout of a river valley is shaped by natural conditions, leading to a restricted expansion of the urban area. Correspondingly, the compact urban space also conforms to the belt-shaped road network design, thus reflecting the future close urban development concept.

The process of urban development frequently results in the creation of road networks in cities located in river valleys, which typically adopt the form of strips or belts [28, 29]. Multiple studies conducted on cities located in river valleys examining various aspects of traffic, road classification, and urban functionality have consistently shown that the majority of urban traffic is concentrated on the main roads that are aligned with the central axis of the metropolitan area. In this context, the road system in the city is typically shaped by the narrow river valley and belt topography, which leads to a scarcity of land for urban development [30]. This has led to various challenges, including the problem of traffic congestion within the city. Furthermore, the uneven distribution of road networks also influences the spatial arrangement and configuration of facilities in river valley cities [31, 32]. In this context, it is worth noting that traffic congestion in river valley cities predominantly occurs in historic urban areas and on bridges that cross the river [33]. These transportation infrastructures in the city bear a substantial portion of the traffic volume; however, the current road network is largely insufficient to accommodate the increasing demand for travel, thereby resulting in severe congestion [34, 35].

The major factors that influence the urban spatial configuration comprise the characteristics of the road network, which facilitate the quantitative analysis of the coupled interaction between various parts of the urban space with each other and with the surrounding areas [36]. In this context, Lammera, Abdulla, and other scholars conducted a study on the traffic network of German cities, specifically analyzing its complexity and small-world characteristics [37, 38]. In addition, a research study investigating the correlation between street networks and traffic flow in the city center revealed that urban road traffic networks can be broadly classified into several types, such as square grid, belt, radial, circular radial, and free-form [39, 40]. However, the physical road infrastructure is not limited to a single configuration, but often consists of a combination of two or more basic road networks [41–43]. Accordingly, Syukri et al., utilized Geographic Information Systems (GIS) to determine the spatial availability of transportation in order to reveal the principles that govern road network accessibility [44]. In this context, spatial syntax is deemed an essential analytical approach used in urban planning and transportation accessibility to examine the complex features of urban networks [45–47]. In urban environments, it frequently functions as a valuable tool for discerning between complete and incomplete spatial areas. Correspondingly, it is employed to depict the clustering and dispersal within the complex system of urban areas, which in turn are interconnected and influence socio-economic activities [48]. The main focus of Spatial syntactic modeling primarily addresses two key issues: the abstract urban morphological structure and the interaction between the physical form of cities and socio-economic activities [49]. Within this framework, Liu et al., carried out a study to validate the topological accessibility and spatial network structure principle of the Wuhan metropolitan area by constructing a spatial axis model [50]. In addition, numerous researchers have conducted a comparative analysis to combine measurements related to the availability and spatial pattern characteristics of road networks in regions with intricate topography [51]. Moreover, multiple studies have substantiated the effectiveness of the spatial syntax approach in analyzing urban morphology, functional organization, and transportation networks, thus offering an objective reflection of the characteristics of complex urban systems [52].

Over the past few years, there has been a persistent use of spatial big data and emerging methods to analyze urban issues. For instance, multiple studies report the use of enterprise distribution data and night lighting data to examine the spatial development characteristics of cultural industries and the changes in urban spatial patterns [53, 54]. Furthermore, the application of multivariate data, such as satellite remote sensing data (including remote sensing imagery, traffic dynamics, and heat maps), has also been documented as a means to assess the traffic pattern and congestion status in urban areas [55]. With the improvement of artificial intelligence and powerful data processing capabilities, cell phone positioning data [56, 57], intelligent card data [58], and cab GPS data [59] have been able to reflect the direction of urban spatial development and internal organization more intuitively. In addition, the utilization of remote sensing data and deep learning techniques [60–62] enables the extraction of road networks, which in turn provides essential data support for urban spatial layout and facility distribution.

The majority of studies on urban spatial morphology concentrate on the correlation between facilities and functions within the city [16, 42, 63], or the expansion of space beyond the city [64]. Nevertheless, existing studies frequently overlook the interaction between internal and external space. The road transportation network is widely acknowledged as the fundamental framework of the city [7, 65]. Accordingly, the spatial and temporal evolution of the urban road transportation network and space provides valuable insights into the mechanism of spatial development in river-valley cities.

The current study offers valuable insights into the correlation between function, land use, and the environment in river valley cities. Herein, nighttime lighting data and historical road network data of Lanzhou, a typical river valley city, was used to analyze and describe the evolution of urban spatial patterns. In this context, a spatial syntactic model was first used to measure the accessibility and evolutionary characteristics of the urban road transportation network over the past 20 years. Based on the resultant dataset, the evolutionary attributes of urban morphology in different periods are depicted in terms of fractal dimension, compactness, and integration. Finally, the coupling and synchronization between the layout of the road network and the morphology of urban areas are validated. These findings provide a crucial basis for the development of urban spaces and the formulation of urban planning strategies. However, despite the extensive analysis, this study was unable to investigate the underlying dynamics of these factors at a more detailed level due to the constraints of the sample data. Future research along these lines is likely to aid in conducting comparisons of multiple river-valley urban spaces and investigating the interaction between urban transportation networks and spatial morphology using microscopic simulation.

## Materials and methods

### Regional overview

**Fig 1:**
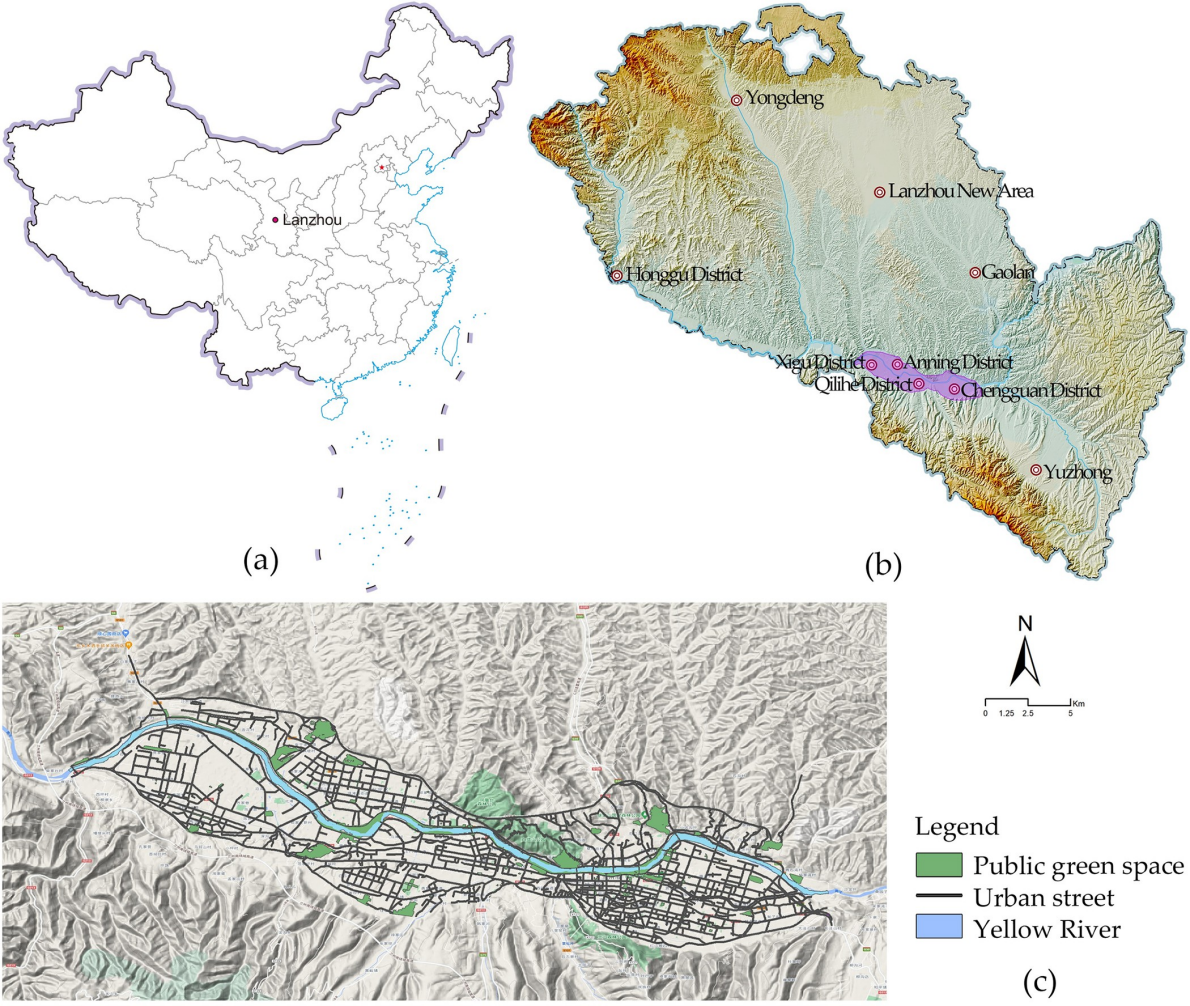
Study area. (a) Location of Lanzhou in China, (b) Topographic and geomorphological features of Lanzhou, (c) Morphology of Lanzhou city center and road network layout.

Lanzhou City is located in the northwestern part of China and is positioned at the geometric center of China’s continental land map. The city’s topography is marked by higher terrain in the western and southern parts, while the northeastern area has lower elevations. The city center is located at the geographical coordinates of 36°03′ north latitude and 103°40′ east longitude. The geographical features of the city include mountain ranges to the north and south, as well as the Yellow River passing through it. This results in a valley basin topography that can be described as "two mountains sandwiching a river." Lanzhou’s economy has experienced substantial growth in recent years, with both the scale and quality of the economy improving. Additionally, the city has seen significant expansion in both its internal and external boundaries (Fig 1).

### Methods

#### Data sources and preprocessing methods

*Road network data*. Three years of historical data (2000, 2010, and 2020) were collected for this study. It included OSM data and China Ministry of Natural Resources (MNR) vector road and river data, which were calibrated using Google historical remote sensing imagery [66]. The transportation infrastructure comprises various components, such as conventional roadways, subway tunnels, and elevated viaducts. Authentic road network data pertaining to the central region of Lanzhou city was obtained through cropping.

*Nighttime lighting data*. The data utilized in this investigation was acquired from the National Geophysical Data Center. More precisely, the data was obtained from the DMSP/OLS and NPP-VIIRS nighttime lighting data [67]. Data was collected at three distinct time points: 2000, 2010, and 2020. The threshold extraction method [68] was employed to determine the optimal lighting value in the nighttime lighting dataset. This was achieved by examining the data acquired from the statistical yearbook. The area that surpassed or matched the lighting threshold was found to be roughly equivalent to the urban built-up area specified in the "China Urban Statistical Yearbook," "Lanzhou Statistical Yearbook," and other relevant sources. As a result, urban areas were identified and defined during different time periods by using a predetermined threshold value.

*POI points of interest data*. The spatial distribution data of Points of Information (POIs) on internal morphological activity points in Lanzhou City for the years 2000, 2010, and 2020 are obtained from Gaode Map. Outliers were eliminated by utilizing coordinate definition and projection techniques, leading to the acquisition of the point data. The present study investigates the outcomes of transformations in the key areas of urban activity; these have been classified into six distinct categories: commercial hubs, lodging and dining establishments, educational and healthcare facilities, public infrastructure (including government institutions and transportation amenities), recreational centers (such as tourism, leisure, and related services), and all socio-economic activities. The data sources are shown in Table 1.

**Table 1:** Source of data.

Type	Data name	Website	Time of collection
**Road network data**	OSM road network data	https://www.openhistoricalmap.org	May 15, 2022
	Road vector data from the website of the Ministry of Natural Resources of China	http://www.mnr.gov.cn	December 10, 2022
**Nighttime lighting data**	DMSP/OLS and NPP-VIIRS nighttime lighting data	https://www.ngdc.noaa.gov/eog	October 5^th^ to October 7^th^, 2022
**Statistical data**	China Urban Statistical Yearbook,	https://www.stats.gov.cn/zs/tjwh	
	Lanzhou Statistical Yearbook	https://tjj.lanzhou.gov.cn/art/2024/1/31/art_4866_1315620.html	
**POI Points of Interest data**	Gaode map Points of Interest Data	https://www.ngdc.noaa.gov/eog	October 15, 2022

**Fig 2:**
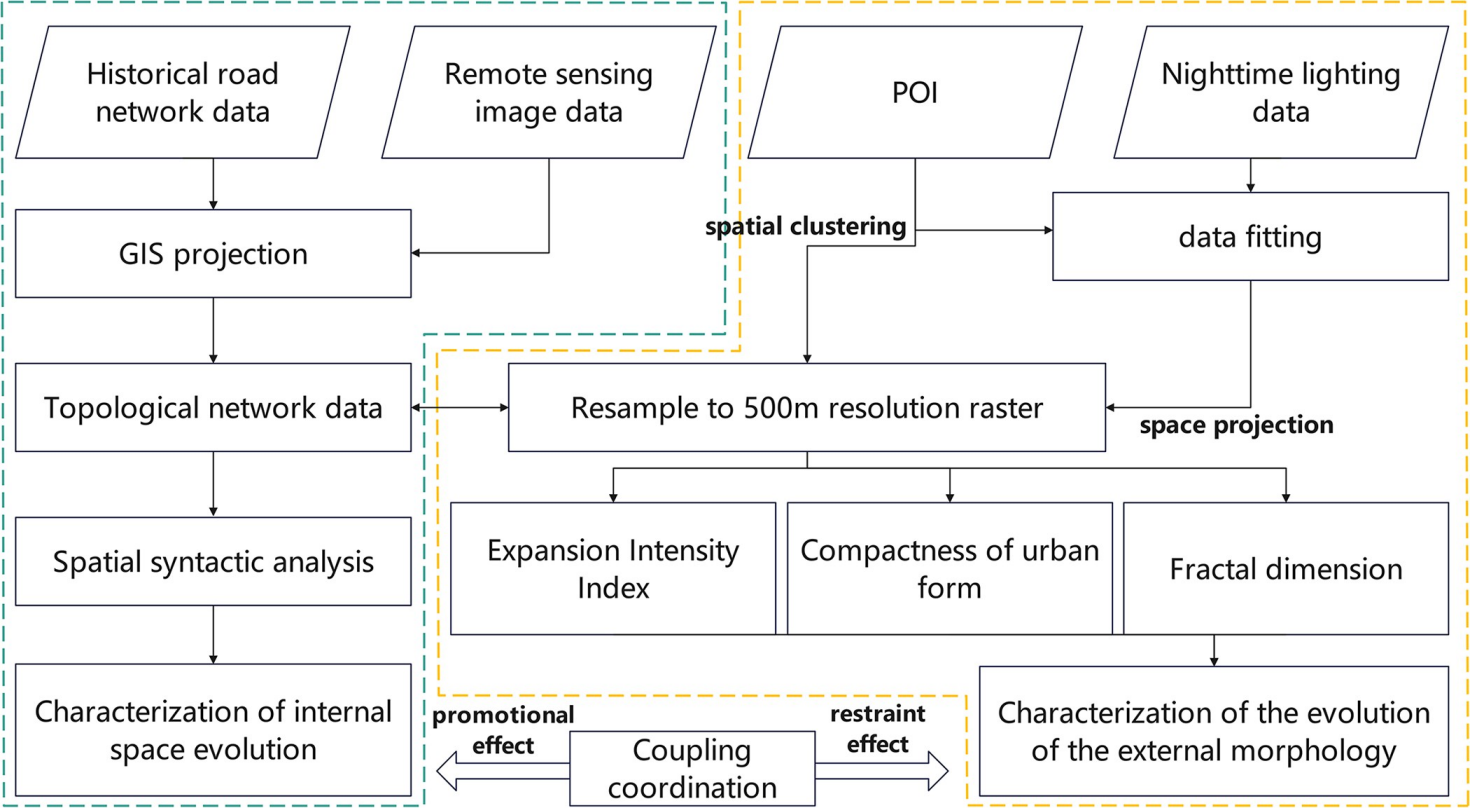
Flowchart depicting the data preprocessing approach used in the current study.

The data preprocessing and process used in this study are illustrated in Fig 2. The initial phase of this study involves aligning the gathered data to a consistent geospatial coordinate system. The ArcGIS software is then used to construct a topological network and spatial clustering, resulting in the generation of vector data that serves as the primary dataset for analysis. Subsequently, the raster with a resolution of 500 meters was resampled and the resulting data were integrated into the same index unit. The data were then analyzed using spatial syntax, extended intensity, morphological compactness, and fractal dimension to obtain specific parameters for the internal and external morphology of the urban space, respectively. Finally, the coupling coordination degree of these parameters is assessed in order to investigate their mutual enhancement and limitation effects.

#### Spatial syntax-based analysis of inner-city morphology

*Degree of integration*. The degree of integration indicates the degree of aggregation and disaggregation of an axis with other axes [69]. In this context, *RRA*_(*n*)_ represents the local integration degree, which indicates the interrelationship between a certain axis and the axes within a few steps (generally three steps) from it. Additionally, *RRA*_(*i*)_ denotes the global integration degree, which indicates the interrelationship between a certain axis and all other axes. When the integration degree is greater than 1, it indicates a high degree of aggregation; on the other hand, when the integration degree is between 0. 4 and 0. 6, it indicates that the axes are relatively dispersed.

$$I_{\left(n\right)}=\frac{1}{RRA_{\left(n\right)}}=\frac{m[\log_{2}(\frac{m+2}{3}-1)+1]}{\left(m-1)|\bar{D}-1\right|},$$ (1)

where, *I* represents the integration of a specified axis in the road composition, which is localized when the value of *N* is 3, *N RRA*_(*n*)_ represents the actual relative asymmetry value, and $\bar{D}$ represents the local average depth value, which is the average of the minimum number of steps from any axis to other axes in the overall composition. The formula is:

$$\bar{D}=\frac{\sum_{d=1}^{s}d\times N_{d}}{m-1},$$ (2)

where, *m* represents the number of axes in the road composition, and *d* represents the shortest step from any axis in the composition to any other axis.

*Synergy*. Synergy refers to the degree of correlation between the local and global integration of the overall network and the regional networks within a specific spatial context. The value indicates that a space with a high level of intellectual capacity has a stronger and more harmonious interaction with the entire network. It also has a greater ability to integrate into the overall growth of the network. Consequently, a reciprocal relationship exists between the two factors, resulting in the increase of specific environments. Urban spatial intelligence refers to the interplay between the overall integration and the localized integration of the axes within a particular area of the road composition structure. The correlation can be mathematically represented by the following formula:

$$R=\frac{{\left[\sum \left(I_{\left(3\right)}-{\bar{I}}_{\left(3\right)})(I_{\left(n\right)}-{\bar{I}}_{\left(n\right)}\right)\right]}^{2}}{\sum {\left(I_{\left(3\right)}-{\bar{I}}_{\left(3\right)}\right)}^{2}\sum {\left(I_{\left(n\right)}-{\bar{I}}_{\left(n\right)}\right)}^{2}}.$$ (3)

In the formula illustrated above, *R* represents the Synergy(comprehensibility) of urban space, represents the value of three-step integration of any axis of space, *I*_(3)_ represents the average value of three-step integration of any axis of space, ${\bar{I}}_{\left(3\right)}$ represents the value of global integration of any axis, and ${\bar{I}}_{\left(n\right)}$ represents the average value of global integration.

#### Characteristics of the evolution of urban external spatial patterns based on nighttime lighting data

*(1) Expansion intensity index*. The urban expansion intensity index is a quantitative measure that calculates the proportion of the increase in built-up area within a specific study area compared to the total area over a defined time period. This index evaluates the extent of urban development expansion across different temporal intervals.


$$M_{ve}=\frac{\Delta U_{nj}}{(\Delta_{tj}\times ULA_{nj})\times 100\%},$$ (4)

where, *M*_*ve*_ represents the rate of urban land expansion, *j* represents the number of active built-up areas in a given direction in the time period, Δ_*tj*_ represents the time span covered in the time period, and *U*_*nj*_ denotes the total area of the district at the beginning of time period *j*.

*(2) Compactness of urban form*. Urban shape compactness refers to the overall development of urban areas, indicating that the transformation of a city’s external boundary is intrinsically linked to modifications in its internal configuration. Therefore, the development of the internal organization of a town will inevitably lead to the enlargement of its external contour. The formula used to calculate the measure of shape compactness is represented below:

$$c=\frac{2\sqrt{\Pi A}}{P},$$ (5)

where, *c* refers to the compactness of the city, *A* refers to the area of the built-up area of the city, *P* refers to the perimeter of the city contour. The value of *c* is between 0 and 1. The larger the value of compactness is, the more compact the shape is; on the contrary, the larger the value of compactness is, the less compact is the resultant shape.

*(3) Fractal dimension*. The fractal dimension is a numerical measure that quantifies the logarithmic correlation between the perimeter of the boundary line of an urban built-up area and the area of the built-up area. It is frequently used as a measure of the spatial pattern complexity of the built-up area [70]. The formula can be expressed as follows:

$$S=\frac{2ln(\frac{P}{4})}{lnA},$$ (6)

where, *S* denotes the fractal dimension value, *P* denotes the perimeter of the built-up area, and *A* denotes the area of the built-up area; the larger the value of *S*, the more complex the shape of the built-up area.

#### Degree of coupling coordination

A comprehensive evaluation index system was established to examine the interplay between the "internal-external" connection and the characteristics of coordinated development. The low values of the two indicators can be ascribed to the disparities in the topological characteristics of the road network and the magnitude of urban spatial expansion. This observation is corroborated by prior research [71, 72]. To adequately address the complex relationship and imbalance between the road transportation network and urban form, it is essential to develop a metric that quantifies the level of road network accessibility and the extent of coordination and integration between the road network and urban spatial patterns. The formula can be expressed as follows:

$$C=[\frac{u_{1}\times u_{2}}{\left(u_{1}+u_{2})(u_{1}+u_{2}\right)}{]}^{1/2},$$ (7)

$$D=\sqrt{C\times T},T={\mathrm{au}}_{1}+bu_{2},$$ (8)

where, *C* is the coupling degree of road network and form, 0 ≤*C* ≤1, the larger the value of *C*, the higher the degree of road network and form coupling; *D* is the coupling degree of coordination between road network and form, the larger the value of *D*, the better the effect of coupling degree of coordination; *T* is the comprehensive coordination coefficient between road network and form; both *a* and b represent a constant quantity due to the change of the road network and the urban form in the process of urban development, which can promote each other. However, numerous influencing factors exist between *a* and b, which play a certain role. Accordingly, *a* and b were assigned 0.4 and 0.6. Additionally, *u*_1_ denotes the standardized road network through the entropy value method of the integrated weight index and weight weighting.

In order to more accurately measure the correlation between the level of road network accessibility and the degree of coupling and coordination in urban form, utilized the classification outlined in Table 2 was utilized in the current study. This classification categorizes the intervals and grades for the level of accessibility of road networks and the degree of coupling and coordination of urban spatial form.

**Table 2:** Classification of coupled coordination types.

Type	Coupling Coordination States	Coupling Coordination Type
Harmonized development	(0.9–1.0)	Perfect coordination
	(0.8–0.9)	Good coordination
	(0.7–0.8)	Intermediate coordination
	(0.6–0.7)	Primary coordination
Transformational development	(0.5–0.6)	On the verge of disorder barely coordinated
	(0.4–0.5)	On the verge of disorder
Uncoordinated development	(0.3–0.4)	Mild disorders
	(0.2–0.3)	Moderate disorders
	(0.1–0.2)	Severe disorders

## Results

### Characterization of structural spatio-temporal evolution of urban road networks

**Fig 3:**
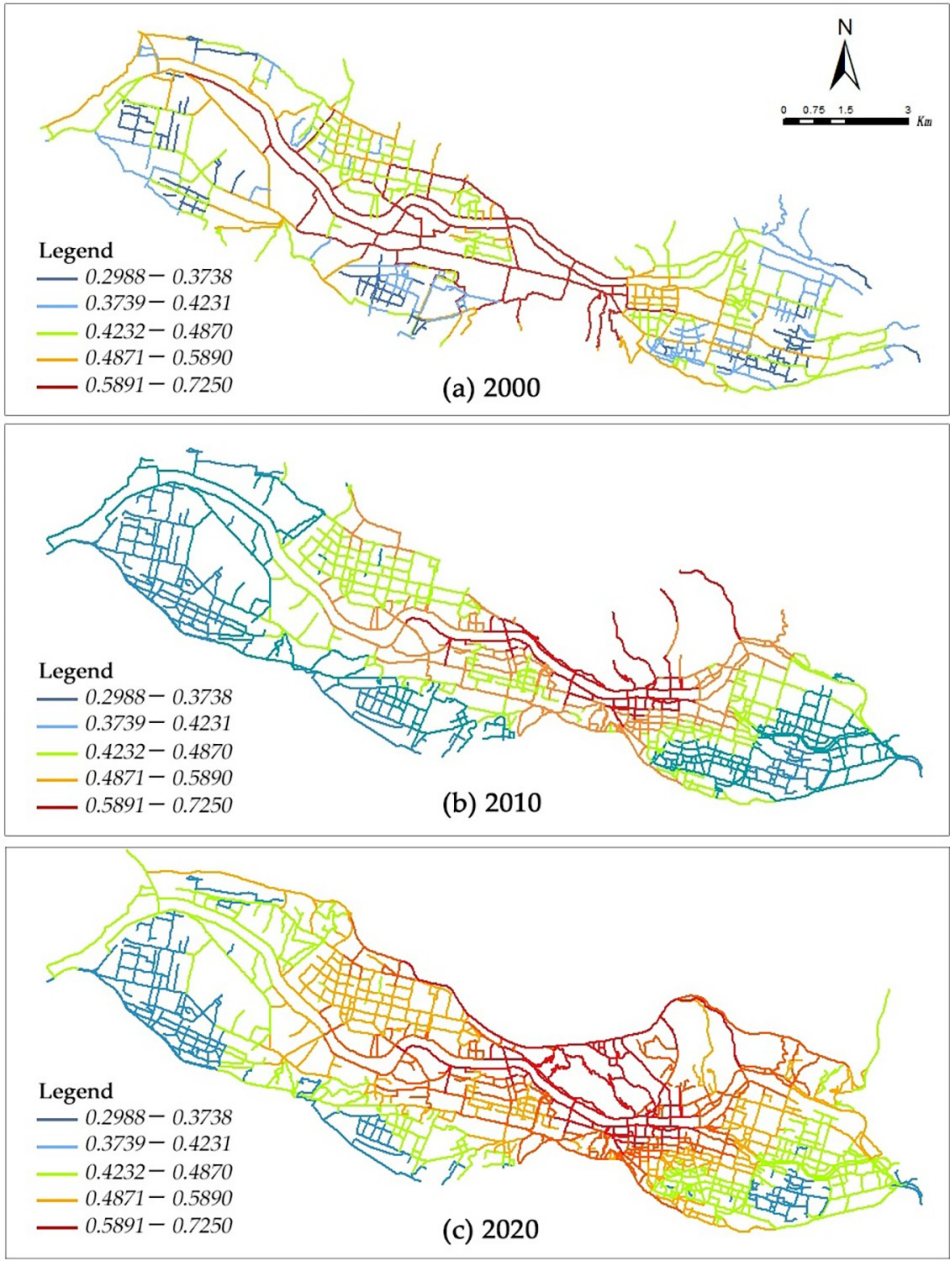
Global integration and evolution of the core area of Lanzhou City in different time periods.

In the year 2000, the central aspect of global integration was characterized by a distinct and restricted range, demonstrating a noticeable spatial organization known as the "banded single core" pattern (Fig 3A). The level of global integration, as measured by the *RRA*_(*i*)_ values, varied between 0.30 and 0.72, with an average value of 0.52. Notably, Xijin West Road demonstrated the highest level of integration (0.72), with Xijin West Road–Xijin East Road (0.71) and Zhongshan Road (0.70) following closely behind. It is worth noting that the axes that had a significant level of global integration were mainly concentrated in the eastern region of Xiguan. More specifically, they were located at the intersection of Xijin West Road and Zhongshan Road. This observation highlights the unique single-core pattern that is clearly visible in the spatial morphology of the city. Additionally, the single-core pattern demonstrated a gradual decline in the core *RRA*_(*i*)_ value as it moved outward. During this temporal phase, a spatial arrangement characterized by a horizontal branching structure of highly valuable global integration axial clustering area emerged, with its center located at Xijin Xilu and east-west Zhongshan Road. This indicates that the urban spatial configuration expanded progressively in the western and eastern directions.

By 2010, the global integration nucleus was likely to experience a small increase, while the east-west integration nucleus axis was predicted to undergo substantial expansion. This is likely to form a spatial pattern characterized by "strip-like primary and secondary nuclei" (Fig 3B). Accordingly, the Rn value, which measures the level of global integration, was found to vary between 0.29 and 1.59, with an average value of 0.68. In this context, the Xijin West Road held the highest value (1.03), followed by the area under Xijin West Road and Xijin East Road (1.02), and Nanbinhe East Road (1.01). The findings, therefore, suggest a significant increase in global integration, as well as a substantial improvement in overall spatial accessibility and connectivity. Furthermore, during this temporal epoch, the focal point of global integration in the city was predominantly concentrated in the Chengguan District, exhibiting a layout similar to a strip-shaped integration belt. Subsequently, this integration belt was observed to progressively extend towards the western regions, eventually surpassing the boundaries of the old city limits. Correspondingly, the large-scale efforts in development, construction, urban renewal, and renovation notably improved the integration of the regional axes. As a result, the trend of global integration was highly pronounced, even on a smaller scale. Thus, the overall spatial pattern consisted of a primary and secondary dual-core pattern, with indications of spatial equilibrium starting to appear.

By 2020, a significant expansion in the scale of the global integration nucleus was anticipated, leading to the emergence of a spatial pattern referred to as the "banded nucleus." Moreover, the level of global integration at the eastern and western boundaries of the banded area was anticipated to become increasingly balanced (Fig 3C). Correspondingly, the global integration degree, as measured by Rn values, was found to vary between 0.25 and 1.21, with an average value of 0.72. The level of global integration increased even further; however, it is worth noting that Zhongshan Road maintained the highest degree of integration (1.21). During this specific timeframe, a substantial increase in the scale and scope of global integration along the central axes was observed. Accordingly, a band-shaped integration core emerged, which was centered around Zhongshan Road, and stretched from Nambin and East Road in the east to Xijin West Road in the west. Furthermore, the central part of this band-shaped area underwent substantial growth in its integration core, resulting in a more prominent state of equilibrium. As a result, a consistent dual-core pattern emerged, influencing the overall spatial arrangement. This could be attributed to the rapid expansion of urban areas and the development of infrastructure in the integrated core region, thus leading to a significant increase in regional integration.

#### Analysis of urban road network synergies

**Fig 4:**
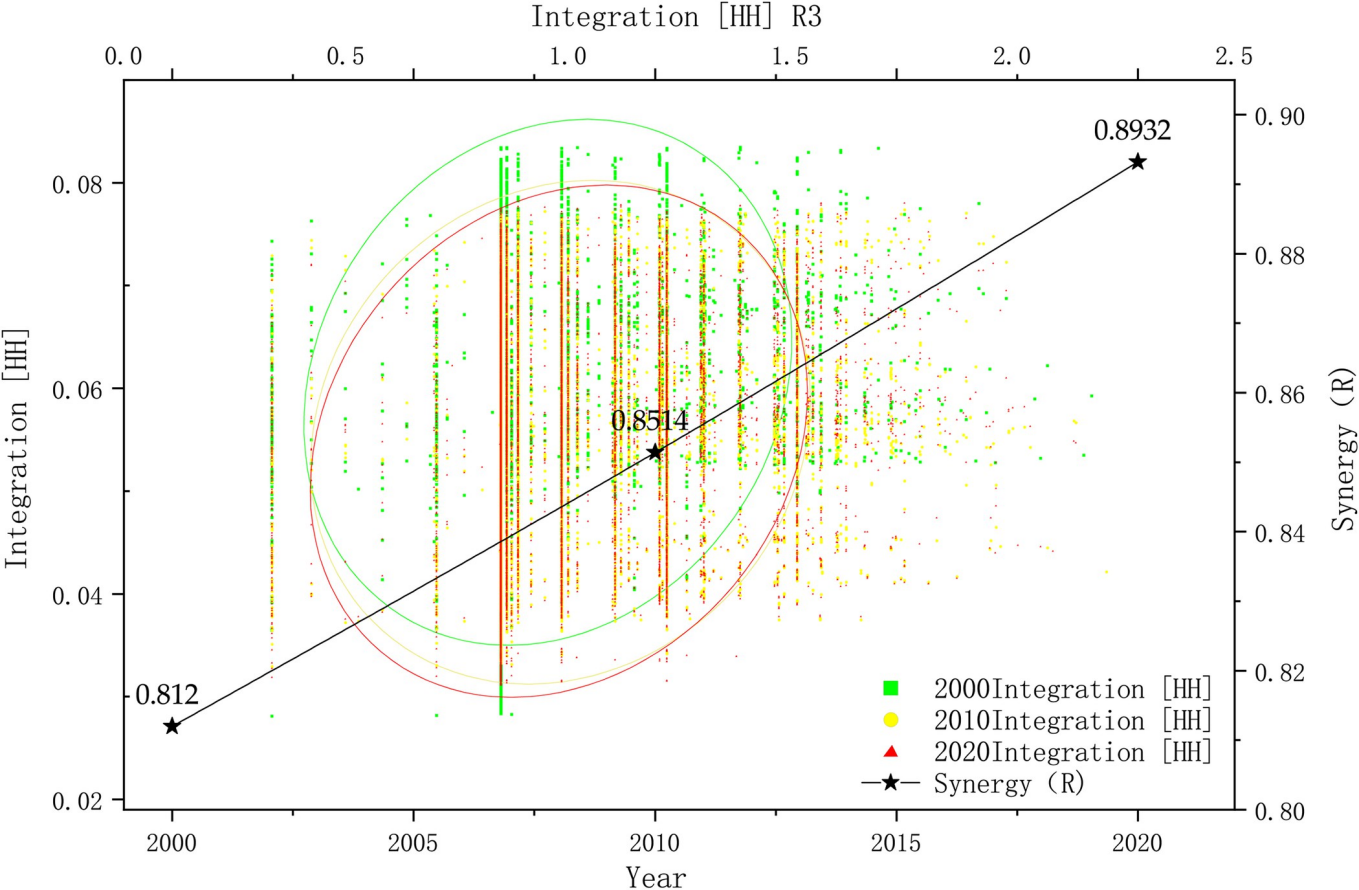
Synergy levels in Lanzhou City during three different time periods.

The scatter plot depicted in Fig 4 demonstrates the spatial synergy of Lanzhou City. The level of comprehensibility in Lanzhou City was measured to be 0.8120, 0.8514, and 0.8932 in the years 2000, 2010, and 2020, respectively. These values exhibited a steady and continuous increase over the years. Furthermore, the degree of synergy in the road network of Lanzhou City was found to be influenced by its spatial environment, characterized by a river valley. This influence was evident in urban areas, where there is a moderate perception of connectivity from local to overall space. Furthermore, at the subdistrict level, there was a stronger perception of connectivity from local to overall space.

Between 2000 and 2020, the growth of the center in Lanzhou City coincided with the road network development in the primary urban area. However, the spatial distribution of the road network exhibited characteristics resembling a river valley belt-like group rather than a cohesive and unified structure like a square lattice network or a circular radial pattern. Consequently, the road network in Lanzhou City’s Chengguan District, Qilihe District, Anning District, and Xigu District developed as separate groups, resulting in weak connections between these groups and an overall lack of balance in the road network. In addition, the quantity of roads with extensive connectivity and regulation could have been augmented, leading to a reduced overall degree of traffic selectivity. Moreover, the road network in Lanzhou City exhibited a lack of strong connections, poor balance, and limited integrity. More precisely, the primary urban road network required a sufficient number of highly connected and controlled roads. Consequently, the overall level of traffic selectivity likely increased. However, horizontal roads demonstrated more robust traffic selectivity compared to vertical roads among the limited high-selectivity roads. This pattern was also evident at the municipal scale in terms of global integration, and at the district scale in terms of local integration. Correspondingly, the global integration at the city scale and regional integration at the district scale could be described as having a "monocentric" configuration, wherein proximity to the central area corresponded to a greater topological accessibility, while greater distance led to a lower topological accessibility.

### Characterization of the evolution of the external spatial pattern of the city

#### (1) Expansion intensity analysis

Between 2000 and 2010, the city of Lanzhou underwent a significant growth rate of 4.95 km2/y, resulting in an expansion intensity of 62.16%. This urban expansion led to a significant increase in the total urban area, indicating a state of rapid expansion and exhibiting distinct stage characteristics. Between 2010 and 2020, the rate of urban expansion reached its lowest point compared to three other time periods, with a measurement of 1.48 km2/y. Moreover, the magnitude of this growth was documented at 18.64%. On the other hand, the current rate of urban space expansion was 18.64%. This stage was classified as part of the "medium intensity and low speed" phase of urban development. The overall growth of the urban area during this stage was relatively limited. Between 2010 and 2020, there was a significant increase in the pace of urbanization. This has increased the expansion intensity from the previous stage, resulting in a value of 36.69%. In addition, the rate of expansion consistently increased, gaining 3.47%. Thus, the current rate of urban expansion, which stands at 3.47 km^2^/y, suggests that the metropolitan area has been experiencing a phase of "low-intensity, high-velocity" expansion. This trend also emphasizes the precise trajectory of urban growth. As observed, the urban function of Lanzhou is being enhanced, and its urban construction is being expedited by establishing Lanzhou New Area as a key growth center in the western region and implementing the urban development strategy of hosting industrial relocation.

#### (2) Extended morphological analysis

During the past twenty years, the developed area of Lanzhou City has exhibited distinct stage characteristics in terms of its fractal dimension and compactness. In addition, the urban morphology and compactness values have demonstrated variations (Table 3). In 2000, the fractal dimension of the urban space in Lanzhou was determined to be 1.076, suggesting a relatively low degree of stability in the urban morphology. Additionally, the analysis revealed that the compactness value was 0.434, indicating a low level of compactness in the urban space. Subsequently, the fractal dimension of the urban space was measured to be 1.067 in 2010, suggesting a greater degree of complexity in the urban morphology. Accordingly, the compactness value was determined to be 0.466, indicating that the urban form predominantly showed expansion towards the outer regions. Moreover, between 2000 and 2010, the urban morphology showed intricate and non-compact attributes. Nevertheless, there was a positive shift in the pattern of urban expansion in comparison to the preceding timeframe. Furthermore, Lanzhou’s overall urban spatial morphology demonstrated a tendency towards compactness and stability. Notably, the city expanded in the direction of the internal Qilihe District, demonstrating the utilization of resources. Thus, the efficiency of utilization was enhanced. Subsequently, the fractal dimension of urban space in Lanzhou increased in 2020, reaching a value of 1.163. This increase in fractal dimension suggests a shift towards a more scattered configuration in the city’s urban morphology. Additionally, the metropolitan area experienced a decrease and subsequent increase in compactness, reaching a value of 0.183. This indicates a decline in the spatial organization of the urban area. As observed, the spatial morphology of Lanzhou exhibited dispersed and non-compact attributes between 2010 and 2020, which was in stark contrast to the preceding timeframe.

**Table 3:** Indicators of urban spatial expansion in Lanzhou between 2000 and 2020.

Expansion Indicator	2000–2020	2000–2010	2010–2020	2000–2020
**Expansion speed (km** ^**2**^ **/years)**	4.95	1.48	3.47	4.95
**Expansion intensity**	62.16%	18.64%	36.69%	62.16%
**Fractal dimension**	1.076	1.067	1.163	1.076
**Compactness**	0.434	0.466	0.183	0.434

#### (3) Shift of spatial center of gravity of urban form

Between 2000 and 2010, Lanzhou City experienced significant urban growth, primarily in the northeastern direction (Table 4). This expansion shifted the spatial centroid by a distance of 228.36 m in the direction of the northeast. Moreover, the angle between the direction of expansion and the due east direction was determined to be 51.49°. Based on the third edition of the Lanzhou City Master Plan (2001–2010), significant and substantial changes in Lanzhou’s economic and strategic positioning, as well as the size and structure of the city’s urban layout were observed. These changes were implemented to efficiently carry out the national development strategy of the western region and complied with urban development requirements. Accordingly, during this stage of urban development, substantial construction projects were carried out in both the eastern and western regions, resulting in what is commonly known as the "first wave of urban construction climax." Notably, the development of the west clusters on the outskirts of the city became more prominent, thus affirming the correlation between the growth of large cities and the need for a comprehensive road network as a crucial element of urban expansion [73, 74].

**Table 4:** Direction and distance of the center of gravity shift of urban form change in Lanzhou.

Particular year	Center of gravity coordinates		Center of gravity travel distance	The direction of transfer	Azimuth
	X	Y			
**2000**	2014095.67	4125433.01	-	-	-
**2010**	2014237.831	4125611.73	228.36	NE	51.49
**2020**	2008341.739	4144086.34	19392.65	NW	72.29

From 2010 to 2020, the city of Lanzhou experienced urban expansion in the northeast direction. Accordingly, the city’s center of gravity shifted approximately 19392.65 m towards the northeast, at an azimuth of 72.29° in the northern direction. Notably, there was a discernible pattern of migration towards Lanzhou New District throughout the study. Correspondingly, the urban construction during this specific period underwent a phase marked by increased activity, commonly referred to as the "second round of climax." Due to the continuous need for urban expansion, the Lanzhou District emerged as a center for diverse high-tech industries after more than twenty years of development. As a result, urban development progressively transitioned towards a spatial development model that integrated different sectors and cities. This transition further facilitated the displacement of the focal point of the urban space.

### The coupling relationship between internal and external city forms

The relationship between road network accessibility and urban form was also analyzed in the current study using a coupling coordination degree model and an evaluation index system. As presented in Table 5, the coordination degree evaluation model incorporated expert scoring and the entropy value method. As observed, the present study aimed to assess the accessibility of the road network and the urban form of each indicator weight. These indicators encompassed integration, synergy, urban external form, expansion intensity, extension speed, compactness, and fractal dimension. Accordingly, the objective was to determine the coefficient for each indicator weight to evaluate road network accessibility and urban form accurately.

**Table 5:** Results of the coupled coordination level of road network accessibility and urban form in Lanzhou urban area.

Districts	Time	U1	U2	C	D	value	Coupling Coordination Type
Chengguan	2000	0.81	0.25	0.4245	0.4743	0.4001–0.50	On the verge of dissonance
	2010	0.89	0.65	0.4939	0.6167	0.6001–0.70	Primary coordination
	2020	0.91	0.82	0.4993	0.6572	0.6001–0.70	Elementary coordination
Qilihe	2000	0.73	0.12	0.3482	0.3847	0.3001–0.40	Mildly dysregulated
	2010	0.61	0.53	0.4988	0.5332	0.5001–0.60	Barely coordinated
	2020	0.92	0.32	0.4376	0.5209	0.5001–0.60	Barely coordinated
Anning	2000	0.31	0.18	0.4821	0.3437	0.3001–0.40	Mildly dysregulated
	2010	0.54	0.58	0.4997	0.5290	0.5001–0.60	Barely coordinated
	2020	0.87	0.76	0.4989	0.6376	0.6001–0.70	Elementary coordination
Xigu	2000	0.43	0.65	0.4895	0.5141	0.5001–0.60	Barely coordinated
	2010	0.25	0.71	0.4389	0.4590	0.4001–0.50	Bordering on dysfunctional
	2020	0.65	0.58	0.4992	0.5541	0.5001–0.60	Barely coordinated

The overall accessibility and external form coordination of Lanzhou city’s road network exhibited a gradual upward trend, transitioning from "on the verge of dissonance" to "not to lose" change. A deeper understanding of the interaction between different levels of accessibility in the region facilitated this transformation. However, the expansion of the city’s connectivity with its surrounding areas has appeared to have been impeded by the inherent geographical features of the mountainous areas, particularly those located in the river valleys. Thus, a slow expansion was observed in certain districts, specifically in Lanzhou City, which could be attributed to the natural characteristics of mountainous valleys, resulting in frequent traffic congestion. Policy support for the expansion of Lanzhou City towards its eastern and northern regions has increased significantly in recent years. This expansion underscores the urgent need to accelerate the development of road networks in these areas in order to enhance transportation services for both production and daily activities. Between 2000 and 2020, there had been a comprehensive examination of the two systems of mixed coordination processes. The prevalence of moderate and mild disorders demonstrated a significant decrease, posing difficulties in achieving primary coordination. As a result, the transition from chaos to organization through mixed adjustment was observed. Thus, Lanzhou City should prioritize the enhancement of urban development in order to foster continuous improvement in the accessibility of its road network and the level of urban form. This involves further enhancing and developing the road topology network to achieve a greater level of excellence. By doing so, Lanzhou City can enhance the integration and synchronization between transportation infrastructure and the urban development system to a higher level of sophistication.

## Discussion

This study examines a representative city located in a river valley and extensively employs various sources of data, including historical road topology network, urban nighttime lighting, and urban POIs, to investigate the interconnectedness between the road network and the physical structure of the city at both internal and external levels. The objective was to reveal the interconnection and synchronization between the "road network and urban spatial form." Accordingly, the development of river valley cities was found to be closely linked to the urban road network. Enhancing the accessibility of urban road networks not only facilitates concentrated urban growth [75, 76] but also highlights the crucial role of road network accessibility in the expansion of urban external form [77]. This validates the theory of compact city development. However, the proposed classification also illustrates the process of organic evacuation in river-valley-type urban spaces, which contributes to the optimization of the layout and restructuring of the internal urban spatial form [17, 78].

Nevertheless, the majority of river valley cities exhibit a relatively low level of internal accessibility within the central city [79]. This phenomenon is reflected in the considerable variation of facilities and traffic accessibility within the main urban area. Accordingly, city managers must improve the level of accessibility by improving the structure of the road network, thereby fostering the concentrated development of urban elements. In order to achieve this objective, the construction of a multi-center urban spatial structure has been proposed in earlier studies to promote synchronized spatial development within a river valley-type city [80]. Alternatively, through examination of the outward spatial growth of Lanzhou City, this study illustrates the progression of the spatial configuration from "belt-shaped single core" to "strip-shaped double core" to "group belt-shaped multi-core," thus demonstrating the transformation of the spatial structure in a river valley city. Accordingly, the evolution of the spatial structure from "ribbon single core" to "strip double core" to "group strip multi-core," demonstrates the coordination between the internal and external spaces of the River Valley city. In this context, establishing an efficient road network system among various functional groups appears crucial to realize this coordination and sustainable development. This point further validates the correlation between the road network and the spatial structure features in river-valley-type cities [81, 82].

While the majority of earlier studies have focused on urban spaces resembling river valleys, its scope is restricted due to limitations in data collection and research methodologies. Thus, the research methodology is likely to be refined and improved further in future studies. The following steps appear crucial towards achieving these objectives. First, the study of the evolution process of the urban road network and urban form should prioritize the examination of the road network layout characteristics. Additionally, the relationship between the road network layout and the urban spatial structure should be thoroughly investigated through comparative analysis of various layout patterns, including square grid, belt, radial, ring radial, and free-form. Furthermore, given the complexity of urban spatial structure, it is imperative to incorporate appropriate indicators from complex networks to more accurately assess the development of urban spatial patterns and layout characteristics. In addition, the use of multivariate spatial data analysis and machine learning also appear crucial in addressing these challenges. Studying multivariate integration is likely to enhance our understanding of the laws and characteristics of river-valley urban space, thus offering a scientific foundation for future urban planning and development.

## Conclusions

This study examined the patterns and characteristics of urban spatial expansion by taking into account the accessibility of road networks. In order to transform a river valley city from a "single core with belt" to a "double core with strip" and eventually to a "multi-core with regiment" spatial structure, the establishment of a suitable road network system that connects each group was deemed crucial. Nevertheless, a lack of effective coordination in urban spatial development was also evident. This stage was found to be primarily influenced by the topological characteristics of the road network and the incorporation and collaboration with areas beyond the city. Consequently, the evolution of urban spatial morphology occurred at different scales and through various mechanisms, each displaying its unique characteristics. This observation also emphasizes the complex spatial arrangement of the River Valley city, providing valuable insights for the design and improvement of urban neighborhood spaces and guiding the implementation of advanced urban planning strategies. Thus, the findings of this study are highly likely to provide a scientific foundation in enhancing the technical and management tools through the utilization of multiple network characterization in future studies. Accordingly, this study serves to provide decision-making bodies in urban development with a more objective foundation for making judgments.
